# The role of 9-*O*-acetylated glycan receptor moieties in the typhoid toxin binding and intoxication

**DOI:** 10.1371/journal.ppat.1008336

**Published:** 2020-02-21

**Authors:** Tri Nguyen, Sohyoung Lee, Yi-An Yang, Changhwan Ahn, Ji Hyun Sim, Tiffany G. Kei, Karen N. Barnard, Hai Yu, Shawn K. Millano, Xi Chen, Colin R. Parrish, Jeongmin Song

**Affiliations:** 1 Department of Microbiology and Immunology, College of Veterinary Medicine, Cornell University, Ithaca, New York, United States of America; 2 Baker Institute for Animal Health, College of Veterinary Medicine, Cornell University, Ithaca, New York, United States of America; 3 Department of Chemistry, University of California, Davis, California, United States of America; 4 Department of Molecular Medicine, College of Veterinary Medicine, Cornell University, Ithaca, New York, United States of America; Gifu University, JAPAN

## Abstract

Typhoid toxin is an A_2_B_5_ toxin secreted from *Salmonella* Typhi-infected cells during human infection and is suggested to contribute to typhoid disease progression and the establishment of chronic infection. To deliver the enzymatic ‘A’ subunits of the toxin to the site of action in host cells, the receptor-binding ‘B’ subunit PltB binds to the trisaccharide glycan receptor moieties terminated in N-acetylneuraminic acid (Neu5Ac) that is α2–3 or α2–6 linked to the underlying disaccharide, galactose (Gal) and N-acetylglucosamine (GlcNAc). Neu5Ac is present in both unmodified and modified forms, with 9-*O*-acetylated Neu5Ac being the most common modification in humans. Here we show that host cells associated with typhoid toxin-mediated clinical signs express both unmodified and 9-*O*-acetylated glycan receptor moieties. We found that PltB binds to 9-*O*-acetylated α2–3 glycan receptor moieties with a markedly increased affinity, while the binding affinity to 9-*O*-acetylated α2–6 glycans is only slightly higher, as compared to the affinities of PltB to the unmodified counterparts, respectively. We also present X-ray co-crystal structures of PltB bound to related glycan moieties, which supports the different effects of 9-*O*-acetylated α2–3 and α2–6 glycan receptor moieties on the toxin binding. Lastly, we demonstrate that the cells exclusively expressing unmodified glycan receptor moieties are less susceptible to typhoid toxin than the cells expressing 9-*O*-acetylated counterparts, although typhoid toxin intoxicates both cells. These results reveal a fine-tuning mechanism of a bacterial toxin that exploits specific chemical modifications of its glycan receptor moieties for virulence and provide useful insights into the development of therapeutics against typhoid fever.

## Author summary

The Gram-negative rod-shaped bacteria *Salmonella enterica* serovar Typhi (*S*. *enterica* serovar Typhi or *S*. Typhi) is the cause of the life-threatening disease typhoid fever. Many millions of people including children under the age of five are affected by this infectious disease. Molecular mechanisms underlying typhoid disease progression and the establishment of chronic infection important for the transmission of this human-adapted pathogen are incompletely understood, but typhoid toxin, one of the virulence factors of *S*. Typhi, is suggested to contribute to these processes. Typhoid toxin consists of three functionally distinct subunits: two enzymatic ‘A’ subunits important for intoxicating host cells after the delivery into host cells and one homopentamer of receptor-binding ‘B’ subunit important for the delivery of the toxin into host cells. Typhoid toxin ‘B’ subunit recognizes specific three-sugar structures decorating the host cell surface, whose terminal sugar called N-acetylneuraminic acid (Neu5Ac) can be present in unmodified or modified forms. The modified Neu5Ac possesses additional chemical groups, with 9-*O*-acetylation being the most frequently found modification in humans. This study analyzes glycan expression profiles of primary tissues and cells associated with typhoid clinical signs, the interface between the toxin and glycan receptor moieties through biochemical and structural approaches, and typhoid toxin intoxication profiles of host cells that are genetically engineered to alter the expression of related glycans. This study reveals a fine-tuning mechanism of a bacterial toxin in exploiting a chemical modification of glycan receptor moieties for virulence and thus provides useful insights into the development of efficacious therapeutics against typhoid intoxication.

## Introduction

*Salmonella enterica* serovar Typhi (*S*. *enterica* serovar Typhi or *S*. Typhi) is a human host-restricted pathogen that is the cause of typhoid fever affecting 10.9 million people worldwide annually [1]. Following the ingestion of contaminated food or water, *S*. Typhi invades the intestinal mucosa and spreads systemically to the liver and spleen, which can result in the acute symptoms of typhoid fever. Depletion of immune cells as known as leukopenia is also a characteristic sign of severe typhoid fever [2–6]. In some instances, individuals affected by typhoid fever suffer long-term neurological complications involving motor function deficits [3, 5, 7–9]. A secreted soluble protein virulence factor known as typhoid toxin appears to contribute to these clinical signs and symptoms during human infection. When administered to laboratory animals, typhoid toxin recapitulates many of the severe acute-phase symptoms of typhoid fever, such as lethargy, malaise, and stupor (the meaning of typhos in Greek), along with leukopenia and neurologic complications [10, 11]. A majority of typhoid fever patients exhibit high titers of anti-typhoid toxin antibodies in their sera [12–15]. Primary human cells and tissues relevant to typhoid clinical signs and symptoms express the specific glycan receptor moieties for typhoid toxin [11], although whether at least some of the glycan receptor moieties are further modified by additional chemical groups has not been revealed.

Typhoid toxin belongs to the bacterial AB toxin family and consists of two enzymatic ‘A’ subunits, CdtB (cytolethal distending toxin A subunit; nuclease) and PltA (pertussis-like toxin A subunit; mono ADP-ribosyltransferase) linked to a homopentamer of receptor-binding ‘B’ subunit PltB (pertussis-like toxin B subunit), resulting in its unique A_2_B_5_ configuration [10]. CdtB is a nuclease that induces DNA damage, host cell cycle arrest, and cell death [16]. PltA is a mono ADP-ribosyl transferase [17], but its role in pathogenesis remains elusive. One role of PltA is its contribution to the formation of the unique A_2_B_5_ configuration of typhoid toxin, by tethering CdtB and PltB together to one toxin complex via disulfide bond and hydrophobic interactions, respectively since there is no direct interaction between CdtB and PltB [10]. PltB recognizes the specific trisaccharide motif as its receptor, N-acetylneuraminic acid (Neu5Ac) α2–3 or α2–6 linked to the underlying disaccharide, galactose (Gal) β1-3/β1–4 linked N-acetylglucosamine (GlcNAc) or glucose [10]. Although this motif can be displayed by various types of glycoproteins and glycolipids on cell plasma membranes, PltB preferentially binds to the trisaccharide motif displayed by multiantennary N-linked glycoproteins providing multiple Neu5Acs simultaneously, as opposed to linear N-linked glycans displaying a single Neu5Ac [11].

Typhoid toxin is produced exclusively by *S*. Typhi located in the *Salmonella*-containing vacuole (SCV) in infected host cells [17]. The toxin within the SCV is immediately encased in small vesicles and exported to the extracellular environment, without having access to the host cell cytoplasm [18]. The secreted typhoid toxin then recognizes the specific glycan receptor moieties displayed on cell plasma membranes, followed by receptor-mediated endocytosis of the toxin into cells, which is an essential process leading to cell intoxication [10]. Typhoid toxin appears to have adapted to glycans predominantly expressed in humans since typhoid toxin does not bind to the otherwise identical glycans terminated by N-glycolylneuraminic acid (Neu5Gc) that many mammals including Chimpanzees express as a major form of sialic acids [19]. Typhoid toxin has tropism to cells expressing multiantennary N-linked glycan receptor moieties terminated in the trisaccharide motif sequence, which include immune cells, brain endothelial cells of arterioles, intestinal and gallbladder epithelial cells [11, 20]. The pentameric configuration of the receptor-binding PltB subunit per toxin and the three glycan binding pockets per PltB monomer play an essential role for multivalent interactions between the toxin PltB and glycan receptor moieties [11, 20].

Neu5Ac can be present in a modified form containing additional chemical groups such as acetyl, sulfo, methyl, and/or lactyl groups, with the *O*-acetylated at C-9 position being the most common modification of Neu5Ac found in human cells, along with the formation of 7,9-*O*-acetylated Neu5Ac due to the migration of the acetyl group between C-7 and C-9 positions [21, 22]. The *O-*acetylation of Neu5Ac can also occur at the C-4 position, resulting in Neu4,5Acs [23, 24]. Therefore, at least two different groups of *O-*acetyl transferases are known to be involved in the *O-*acetylation of sialic acids. One is acetyl-CoA:sialate 7(9)-*O-*acetyltransferase (also known as *CASD1*); the other is acetyl-CoA:sialate 4-*O-*acetyltransferase [24], although the gene encoding the latter enzyme has not been identified. Moreover, whether human cells encode 4-*O*-acetyltransferase is elusive [25, 26]. 9-*O*-acetylated (as well as 4- and 7-*O-*) and non-modified Neu5Ac naturally occur as part of host cell developmental and/or homeostasis processes, among others [21]. Some viral pathogens including influenza virus C and coronaviruses that use that modified glycan receptor for their entry into host cells have evolved to exploit such chemical modifications of Neu5Ac for their core life cycles [26–28]. However, thus far, whether bacterial toxins or virulence factors can use similar tactics for their pathogenesis is unknown. Here we investigated specifically on the role of 9-*O*-acetyl modification (the most abundant *O-*acetylated form of sialic acids in humans) of the glycan receptor moieties in the binding and intoxication for *Salmonella* typhoid toxin.

## Results

### Host cells relevant to typhoid toxin-mediated clinical signs express both unmodified and 9-*O*-acetylated glycan receptor moieties

**Fig 1:**
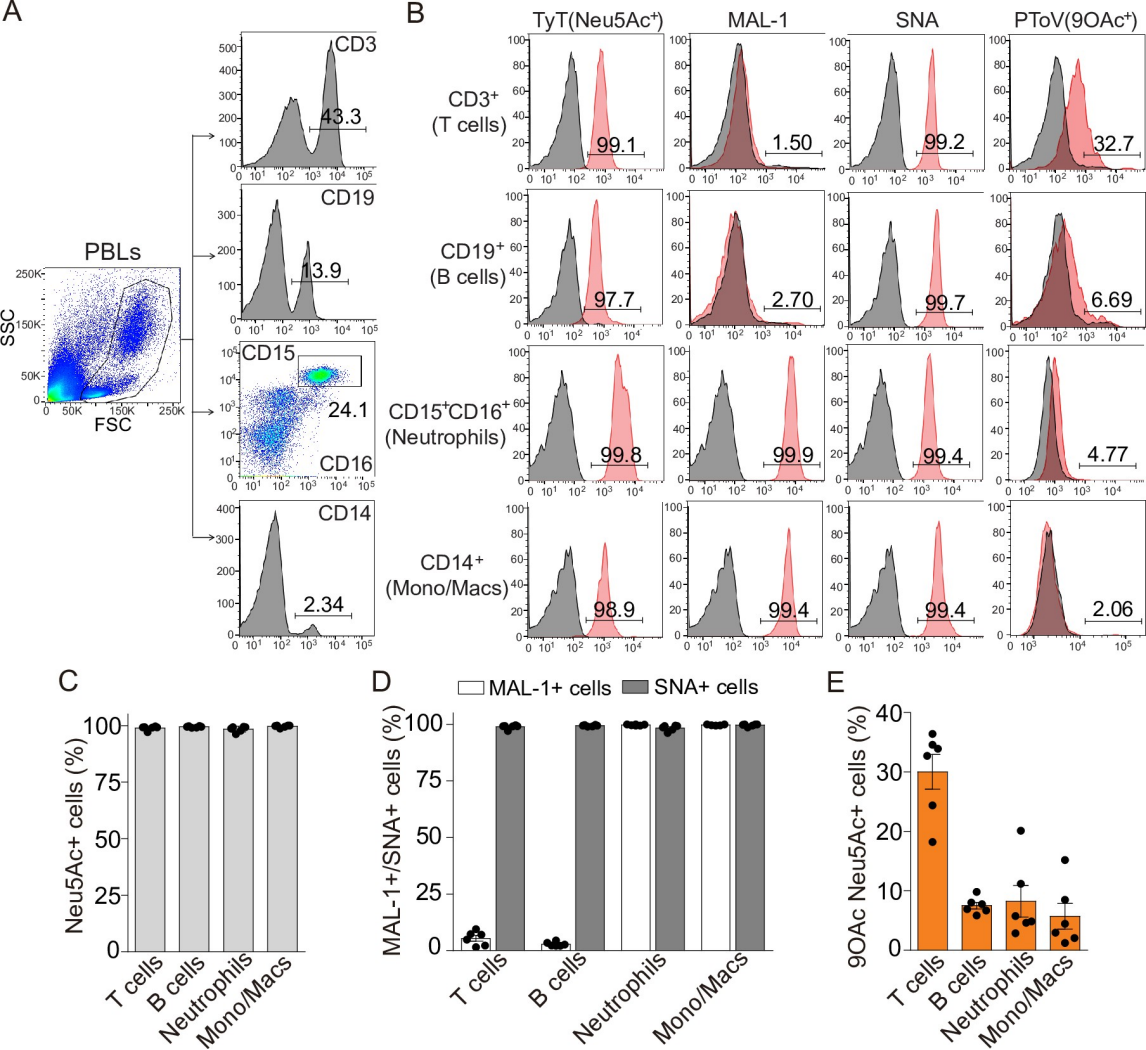
Human PBLs express both unmodified and 9-*O*-acetylated glycan receptor moieties. **A,** Total human PBLs were stained with an antibody cocktail containing antibodies allowing for detecting indicated immune cell subtypes and indicated glycans. Numbers in the plots indicate the percentages of CD3^+^ (T cells), CD19^+^ (B cells), CD15^+^CD16^+^ (neutrophils), and CD14^+^ (monocytes and macrophages) cells. **B,** Representative flow cytometric analysis of indicated glycan expression profiles of hPBLs. Numbers in the plots indicate the percentages of CD3^+^ (T cells), CD19^+^ (B cells), CD15^+^CD16^+^ (neutrophils), and CD14^+^ (monocytes and macrophages) cells that display specific glycans on their cell surface that are recognized by TyT, MAL-1, SNA, or PToV. **C-E,** Neu5Ac expressions (left panels in B and C) on cell plasma membranes of human PBLs were analyzed via flow cytometry. Surface displayed glycans terminated in Neu5Ac in indicated immune cell types were split into two groups based on their linkage between the terminal Neu5Ac and the underlying disaccharide: MAL-1^+^ for α2–3 sialosides and SNA^+^ for α2–6 sialosides (middle panels in B and D). 9-*O*-acetylation of the terminal Neu5Ac was identified by incubating hPBLs with an antibody cocktail allowing for detecting indicated immune cell types and PToV P4 HE premixed with a secondary antibody conjugated to AF488 (right panels in B and E). n = 6 per group.

We first investigated whether host cells relevant to typhoid toxin-mediated clinical signs express both unmodified and 9-*O*-acetylated glycan receptor moieties. When the toxin is administered systemically into mice, typhoid toxin displays tropism to immune cells and brain endothelial cells of arterioles [11]. This toxin tropism is primarily due to the abundant expression of multiantennary N-linked glycans terminated in the trisaccharide motif (Neu5Ac-Gal-GlcNAc) that typhoid toxin recognizes. This multivalent interaction results in high-affinity bindings as indicated in the glycan microarray results allowing for comparisons of their binding affinities [11]. Epithelial cells of the intestine and the gallbladder also predominantly express multiantennary N-linked glycans [20]. In N-linked glycans, the terminal Neu5Ac can be linked to the underlying Gal-GlcNAc via either α2–3 or α2–6 [21]. We found that human lymphocytes predominantly express Neu5Ac α2–6 linked Gal-GlcNAc (to refer the trisaccharide motif terminated in Neu5Ac, we also use the term α2–6 sialosides interchangeably in this manuscript), while human myeloid cells express both α2–3 and α2–6 sialosides, as assessed by MAL-1 and SNA binding to cell surface membranes, respectively (Fig 1A–1D). Human peripheral blood leukocytes (PBLs) were obtained from six volunteers for this analysis. We next evaluated 9-*O*-acetylation of Neu5Ac on cell plasma membranes of human PBLs. Since the porcine torovirus hemagglutinin esterase (PToV-P4 HE) primarily recognizes 9-*O*-acetylated Neu5Ac [25], we exploited PToV-P4 HE that contains an esterase catalytic mutation to assess the expression of 9-*O*-acetylated Neu5Ac on cell plasma membranes and found that 10~30% hPBLs express 9-*O*-acetylated Neu5Ac on their cell surface (Fig 1B and 1E).

**Fig 2:**
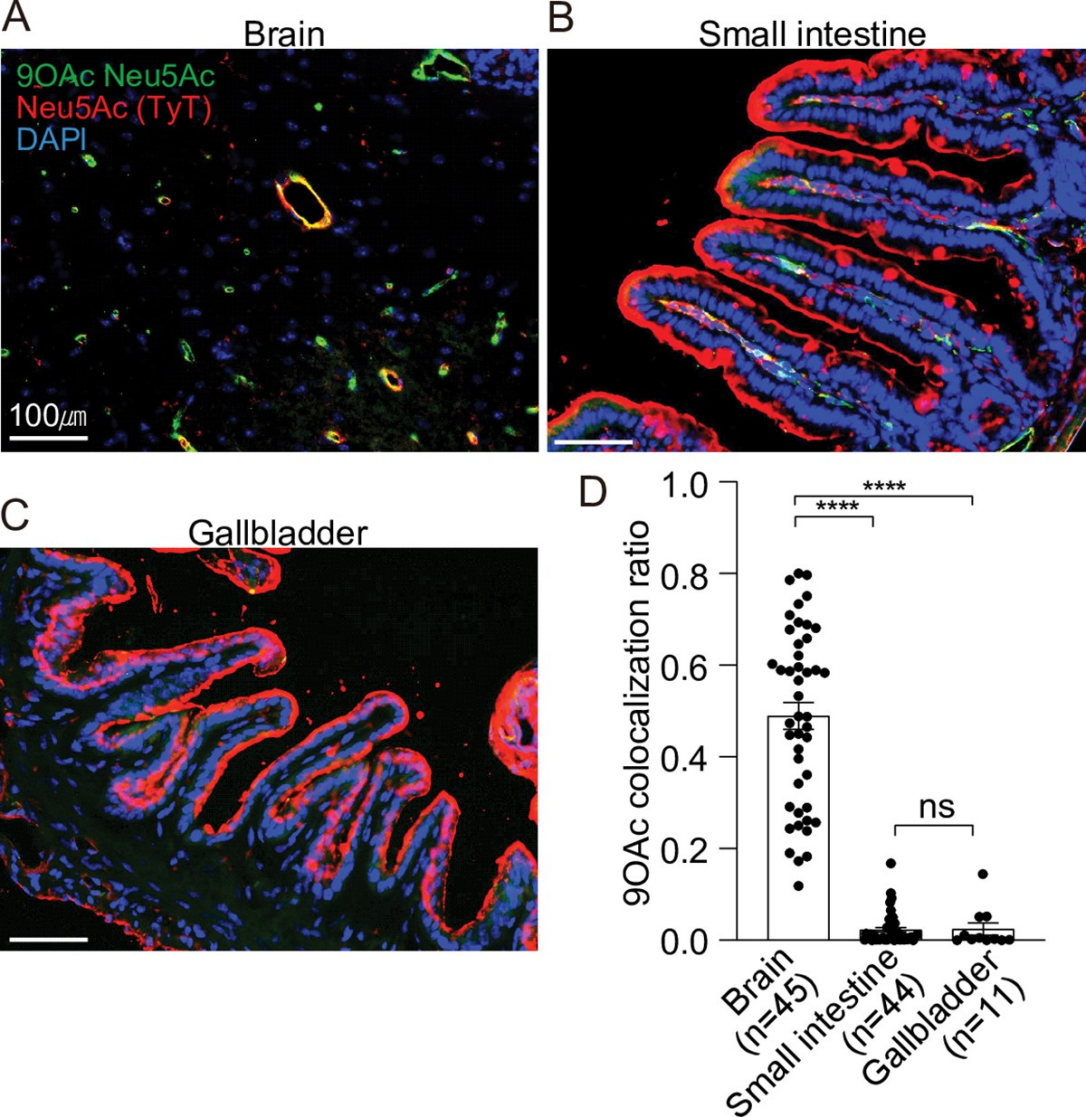
CMAH null mouse tissues and cells relevant to typhoid toxin-mediated clinical signs express both unmodified and 9-*O*-acetylated glycan receptor moieties. **A-C,** Staining of Cmah null mouse brain (A), small intestine (B), and gallbladder (C) tissue sections. PToV-P4 HE-Fc (+ 2^nd^ Ab conjugated to AF488) and typhoid toxin (TyT)-AF555 were used to detect 9-*O*-acetylated Neu5Ac (green) and Neu5Ac (red), respectively. DAPI was used to stain DNA (blue). Scale bar, 100 μm. Representative images used for co-localization quantification are shown. **D,** Co-localization frequency of cells that are positive for both TyT (for both unmodified and modified Neu5Ac) and PToV-P4 HE (9-*O*-Ac Neu5Ac). Y-axis values are the fraction of red signal overlapping green signal. Three independent experiments were performed. Bars represent average ± standard error of the mean. **** p<0.0001. n = 11–45, as indicated in the graph. Two-tailed unpaired t-tests.

We next evaluated the presence of 9-*O*-acetylated Neu5Ac on brain endothelial cells and epithelial cells of the small intestine and the gallbladder. Since cytidine monophospho-N-acetylneuraminic acid hydroxylase (CMAH) null mice exclusively express human-type sialic acid Neu5Ac [19], we used corresponding tissues of CMAH null mice. CMAH is the enzyme responsible for converting Neu5Ac to Neu5Gc in many mammals. However, like CMAH null mice, humans do not express the functional CMAH [29], thus expressing only Neu5Ac. We found that brain endothelial cells of arterioles express a high level of 9-*O*-acetylated Neu5Ac, as assessed by the colocalization of typhoid toxin (recognizing all Neu5Ac) and PToV-P4 HE (recognizing 9-*O*-acetylated Neu5Ac) (Fig 2A and 2D). However, unlike immune and brain endothelial cells of arterioles, we did not observe 9-*O*-acetylated Neu5Ac on the epithelial cells of the small intestine or the gallbladder (Fig 2B–2D). These results indicate that host cells relevant to leukopenia and neurological complications express glycan receptor moieties containing both unmodified and 9-*O*-acetylated Neu5Ac. Among the 9-*O*-acetylated glycans, depending on cell types, the linkage between the terminal Neu5Ac and the underlying disaccharide Gal-GlcNAc is α2–3 and/or α2–6.

### The role of 9-*O*-acetyl modification in the typhoid toxin binding

**Fig 3:**
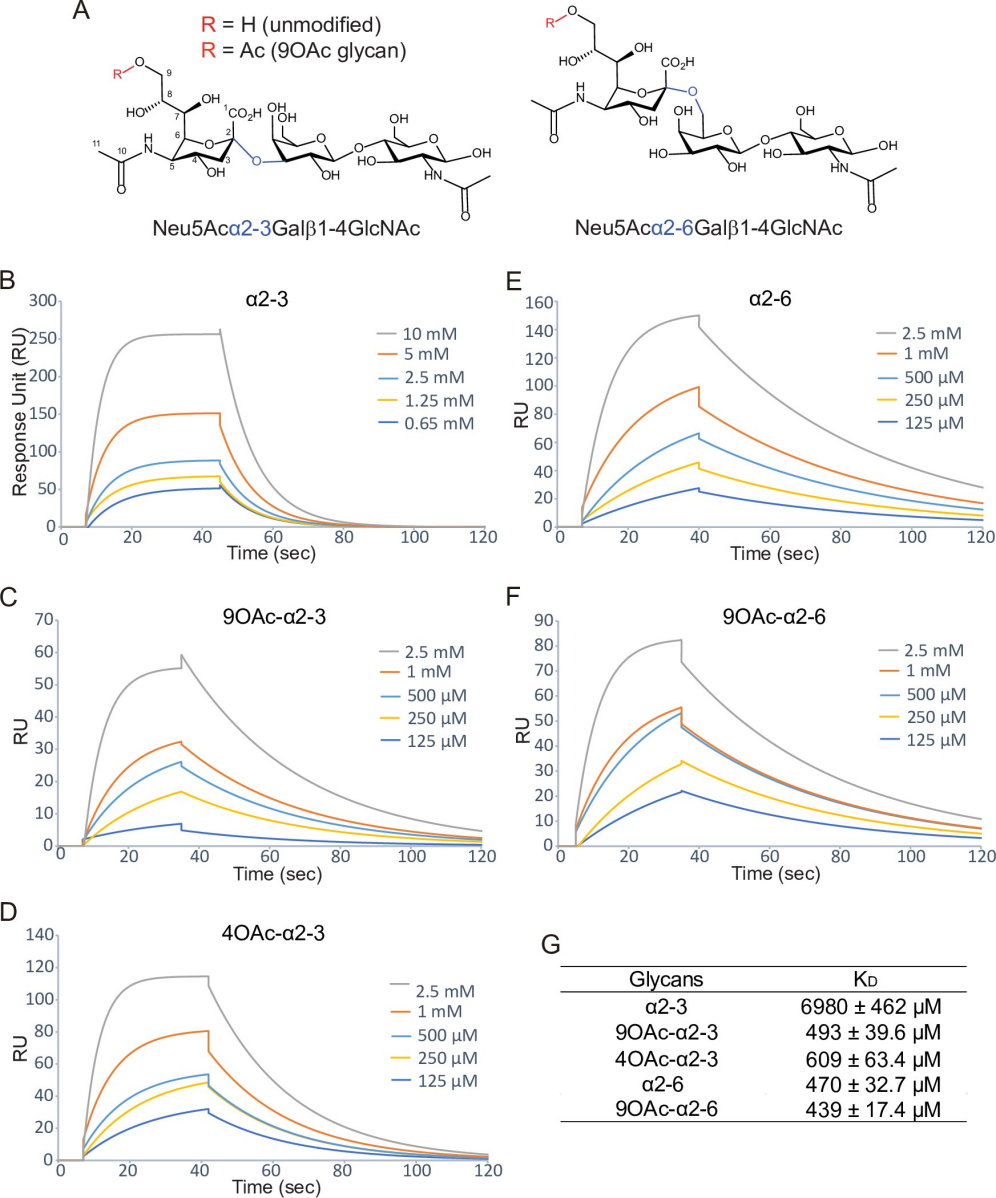
The role of 9-*O*-acetyl modification in the typhoid toxin binding. **A,** Chemical structures of α2–3 and α2–6 trisaccharide motif are shown. R is H in unmodified glycans or Ac in 9-*O*-acetylated glycans. **B-G,** Shown are sensograms (B-F) and K_D_ values (G) of PltB pentamers incubated with various concentrations of indicated glycans.

To evaluate the role of 9-*O*-acetyl modification in the toxin binding, we first synthesized 9-*O*-acetylated α2–3 and α2–6 sialosides, Neu5,9Ac_2_α2-3Galβ1-4GlcNAc and Neu5,9Ac_2_α2-6Galβ1-4GlcNAc, using a one-pot two-enzyme sialylation system [22]. We then carried out surface plasmon resonance (SPR) analysis of these glycans, along with their unmodified counterparts (Fig 3A). We immobilized PltB homopentamer on SPR chips and probed with various concentrations of indicated glycans, to evaluate whether there are any binding affinity differences among them. PltB binds to 9-*O*-acetylated α2–3 glycans (Neu5,9Ac_2_α2-3Galβ1-4GlcNAc) with a ~14-fold increased affinity compared to its unmodified α2–3 counterpart (Neu5Acα2-3Galβ1-4GlcNAc) (Fig 3B, 3C and 3G), while PltB binds to 9-*O*-acetylated α2–6 glycans (Neu5,9Ac_2_α2-6Galβ1-4GlcNAc) with a slightly higher affinity than its binding to unmodified α2–6 counterpart (Neu5Acα2-6Galβ1-4GlcNAc) (Fig 3E, 3F and 3G). It is important to note that the equilibrium dissociation constant (K_D_) values for unmodified and 9-*O*-acetylated Neu5Acα2-3/α2-6Galβ1-4GlcNAc present in the context of multiantennary N-linked glycans, which are more relevant to physiological conditions, are likely much higher than the K_D_ values for the core trisaccharide motif that we assessed in this study. Nonetheless, the results presented in Fig 3 indicate that typhoid toxin binds to 9-*O*-acetylated glycan receptor moieties with increased affinities, compared to its binding to unmodified counterparts. The effect of 9-*O*-acetyl modification on altering binding affinities is more drastic when the terminal Neu5Ac is linked to the underlying disaccharide Gal-GlcNAc via α2–3.

### Structure comparison analysis of PltB subunits bound to unmodified and 9-*O*-acetylated glycan receptor moieties

**Fig 4:**
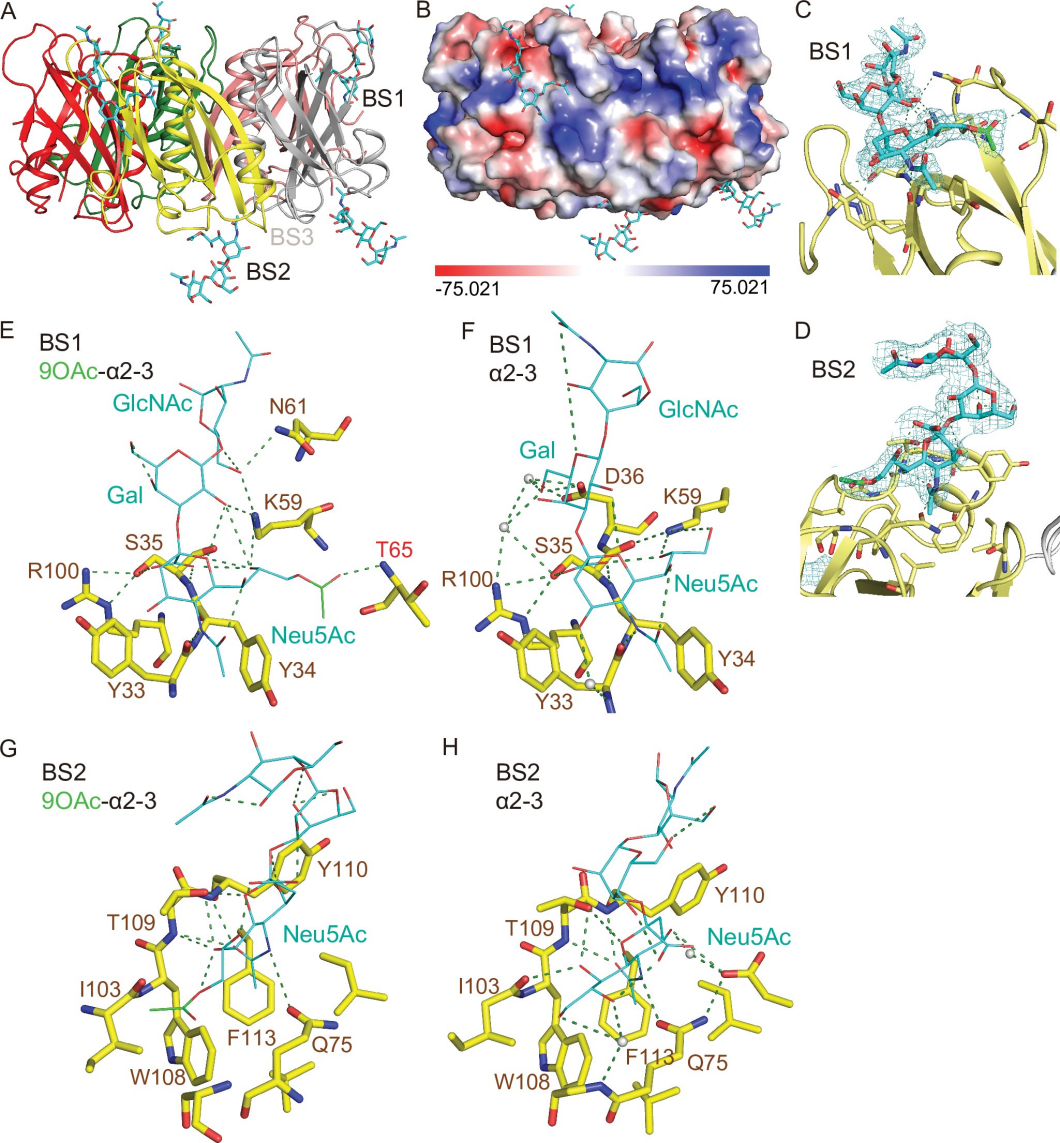
Co-crystal structure of *S*. Typhi PltB homopentamer bound to 9-*O*-acetylated α2–3 sialosides. **A,** Crystal structure of PltB homopentamer in complex with Neu5,9Ac_2_α2-3Galβ1-4GlcNAc is shown as a ribbon cartoon with each protomer depicted in a different color. Cyan sticks, sugar carbon atoms. Blue sticks, nitrogen atoms. Red sticks, oxygen atoms. BS1-3, binding sites 1–3. **B,** Surface charge distribution of the PltB pentamer structure and the indicated glycan. **C-D,** Close-up view of the interface between the BS1 (C)/ BS2 (D) and the indicated glycans with their electron density maps. Green sticks, sugar carbon atoms of the 9-*O*-acetyl group. **E-F,** Close-up views of the BS1 complexed with Neu5,9Ac_2_α2-3Galβ1-4GlcNAc (E) or Neu5Acα2-3Galβ1-4GlcNAc (F). Dotted lines, H-bonds. Gray balls, water. **G-H,** Close-up views of the BS2 with 9-*O*-Ac α2–3 glycan (G) or unmodified α2–3 glycan (H).

**Fig 5:**
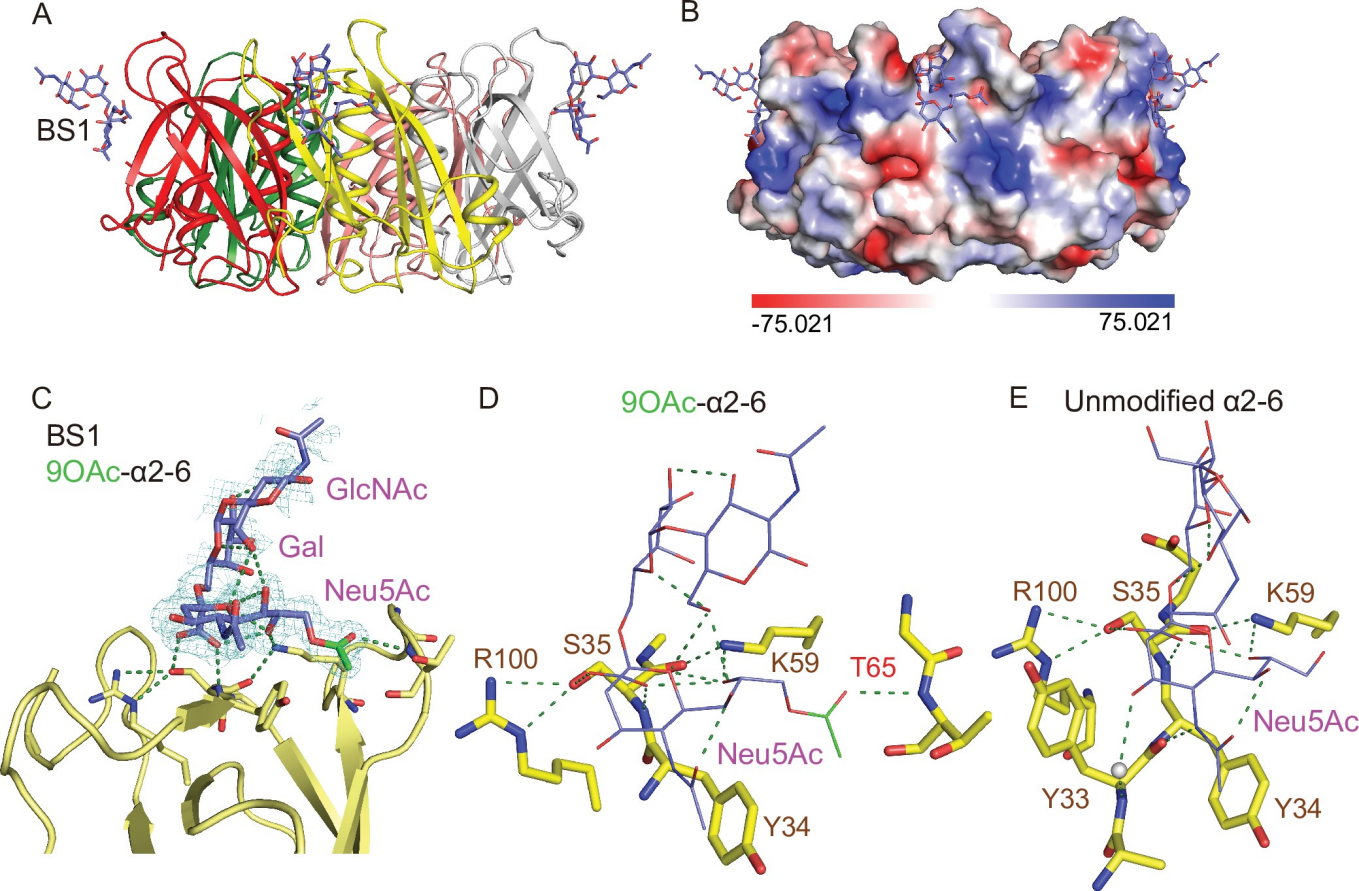
Co-crystal structure of *S*. Typhi PltB homopentamer bound to 9-*O*-acetylated α2–6 sialosides. **A,** Crystal structure of PltB homopentamer in complex with Neu5,9Ac_2_α2-6Galβ1-4GlcNAc is shown as a ribbon cartoon with each protomer depicted in a different color. Purple sticks, sugar carbon atoms. Blue sticks, nitrogen atoms. Red sticks, oxygen atoms. BS1, binding site 1. **B,** Surface charge distribution of the PltB pentamer structure and the indicated glycan. **C,** Close-up view of the interface between the BS1 and the indicated glycan with the electron density map. Green sticks, sugar carbon atoms of the 9-*O*-acetyl group. **D-E,** Close-up views of the BS1 complexed with Neu5,9Ac_2_α2-6Galβ1-4GlcNAc (D) or Neu5Acα2-6Galβ1-4GlcNAc (E). Dotted lines, H-bonds.

We previously revealed the three glycan-binding sites (BS) per PltB monomer and demonstrated the flexible use of these three binding sites in accommodating glycan receptor moieties that are structurally different [20]. For instance, Neu5Acα2-3Galβ1-4GlcNAc (α2–3 sialosides) can use all three binding sites of PltBs, BS1-3, while α2–6 sialosides use only one binding site BS1 located at the lateral side of PltBs. BS2 and 3 are located next to each other at the bottom side of PltB homopentamer (Fig 4A). Key residues include Ser35 and Lys59 for the BS1, Trp108, Thr109, and Phe113 for the BS2, and Asp28 and Asp29 for the BS3 [20]. To gain insight into the underlying mechanism behind the binding affinity difference between 9-*O*-acetylated and unmodified glycan receptor moieties, we solved co-crystal structure of PltB homopentamers bound to Neu5,9Ac_2_α2-3Galβ1-4GlcNAc and Neu5,9Ac_2_α2-6Galβ1-4GlcNAc, respectively (Figs 4 and 5, and Table 1).

**Table 1:** Related to Figs 4–6. X-ray data collection and refinement statistics for the *S*. Typhi PltB co-crystal structures.

	PltB WT with 9OAc-α2–3 glycan	PltB WT with 4OAc-α2–3 glycan	PltB WT with 9OAc-α2–6 glycan
PDB ID	6TYN	6TYO	6TYQ
Glycan bound	3 in BS1 2 in BS2	2 in BS1 3 in BS2	3 in BS1
Date collected	3/15/2019	3/15/2019	7/14/2019
**Data collection**			
Space group	P2_1_2_1_2_1_	P2_1_2_1_2_1_	P2_1_2_1_2_1_
Cell dimensions			
*a*, *b*, *c* (Å)	68.67, 97.75, 101.45	68.58, 98.21, 104.60	69.75, 98.92, 99.63
α, β, γ (°)	90, 90, 90	90, 90, 90	90, 90, 90
Resolution (Å)	40.00–2.33 (2.39–2.33)	40.00–2.04 (2.09–2.04)	99.63–1.88 (1.93–1.88)
*R*_sym_ or *R*_merge_	9.7% (52.6%)	7.1% (14.8%)	12.7% (67.4%)
*I* / σ*I*	37.4 (4.6)	27.79 (4.83)	9.8 (1.3)
Completeness (%)	99.6% (97.0%)	99.8% (99.4%)	98.5% (81.4%)
Redundancy	4.4 (4.7)	6.3 (5.3)	6.1 (2.5)
**Refinement**			
Resolution (Å)	40.00–2.33 (2.39–2.33)	40.00–2.04 (2.09–2.04)	99.63–1.88 (1.93–1.88)
No. reflection	27512 (2036)	45598 (3219)	55467 (3250)
*R*_work_ / *R*_free_	23.10% / 29.86% (27.55%/36.33%)	16.07% / 19.76% (15.80%/20.80%)	19.05% / 21.71% (28.13%/29.86%)
No. atoms			
Protein	4675	4430	4430
Ligand/ion	245	245	147
Water	36	398	177
*B*-factors			
Protein	38.53	15.19	22.09
Ligand/ion	69.79	44.37	45.62
Water	41.74	22.55	25.91
R.m.s. deviations			
Bond lengths (Å)	0.009	0.008	0.007
Bond angles (°)	1.179	1.072	0.993

* Each dataset was collected from a single crystal.

* Values in parentheses are for highest-resolution shell.

BS, binding site.

We found that the overall usage of the glycan-binding sites in PltB homopentamer for 9-*O*-acetylated glycan receptor moieties is similar to their usage for unmodified counterparts. 9-O-acetylated α2–6 glycans bound only to the BS1, while 9-*O*-Ac α2–3 glycans bound to the BS1 and BS2 (Figs 4A, 4B, 5A and 5B). Similar to PltB pentamer bound to unmodified glycan receptor moieties [20], we did not observe conformational changes of PltB pentamer bound to *O-*acetylated counterparts, compared to PltB protomer of typhoid holotoxin (S1 Movie). In close-up views of the interface between the PltB binding pockets and the glycans, we found that there are more interactions between PltB and 9-*O*-acetylated α2–3 glycans than between PltB and unmodified counterparts. Most notably, the 9-*O*-acetyl group forms an H-bond with Thr65 (via the main chain of Thr65) in the BS1, which is absent in the interface of the BS1 bound to unmodified α2–3 glycans (Fig 4C, 4E and 4F). Likewise, Lys59 and Arg61 form H-bonds with Gal and GlcNAc of the receptor moieties respectively, which are also absent in the interface between PltB and unmodified counterparts (Fig 4E and 4F). Water-mediated interactions in the BS1 via Tyr33, Asp36, and Arg100 to unmodified α2–3 glycans were not observed in the BS with 9-*O*-acetylated α2–3 glycans, presumably due to the additional three H-bonds on the other side of the binding pocket that make Tyr33, Asp36, and Arg100 out of reach. Overall, the three additional direct interactions between Thr65, Lys59, and Asn61 and all three saccharides of the glycan receptor moieties correlate to the markedly increased binding affinity of PltB to 9-*O*-Ac α2–3 glycans (Fig 4E). In contrast, only the terminal sialic acid Neu5Ac of unmodified glycan receptor moieties directly interacts with residues in the BS1 (Fig 4F). The interfaces of the BS2 with unmodified and 9-*O*-acetylated glycans were overall comparable, but with the 9-*O*-acetyl group making a noted accommodation through a hydrophobic interaction with Ile103 residue in the BS2 (Fig 4G and 4H). With regard to the PltB BS3 that has been shown previously to bind to Neu5Acα2-3Galβ1-4GlcNAc, the 9-*O*-acetylated glycan counterpart did not bind to the BS3 in either 30 min or 2 hr soaking conditions, respectively (Fig 4A). This appears to be caused by the 9-*O*-acetyl modification presence in proximity to the steric sensitivity area around Asn29 of the BS3, demonstrated previously by loss of binding of unmodified α2–3 sialosides to the BS3 observed in an N29K PltB mutation [20]. Taken together, these results indicate that the markedly increased affinity of PltB to Neu5,9Ac_2_α2-3Galβ1-4GlcNAc compared to the affinity of PltB to Neu5Acα2-3Galβ1-4GlcNAc appears to be due to the increased interactions between the PltB BS1 and 9-*O*-acetylated α2–3 glycans.

**Fig 6:**
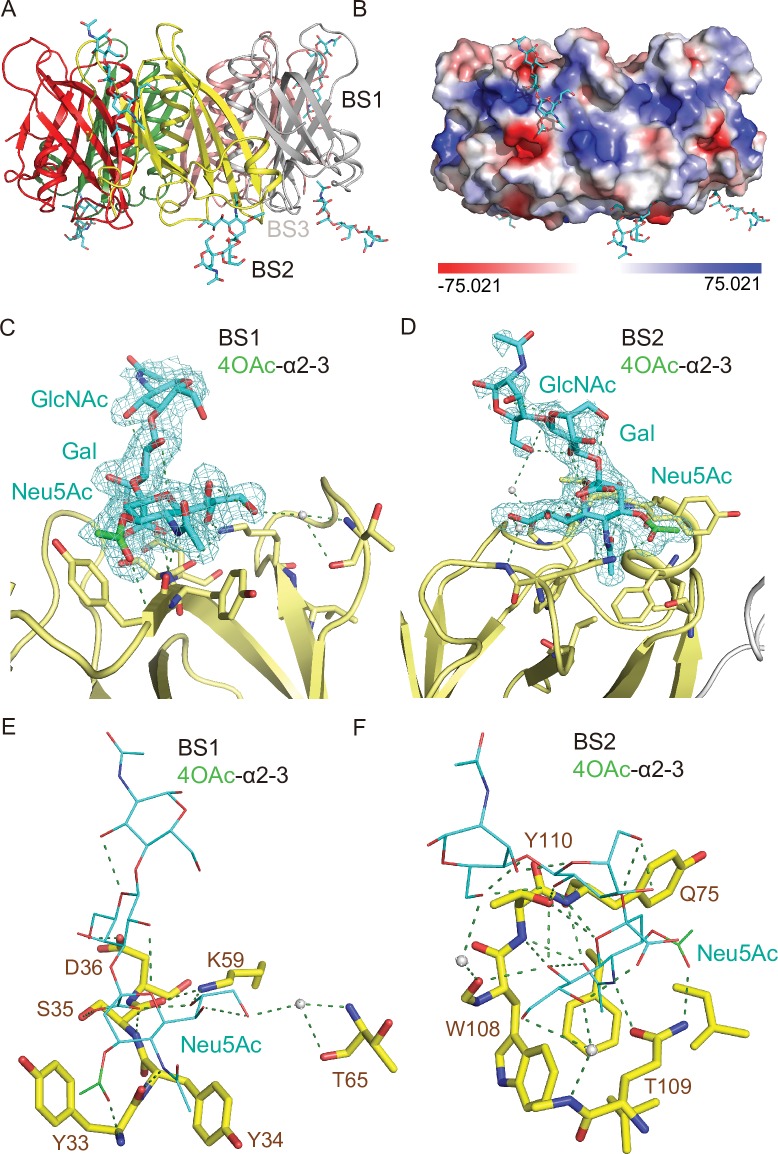
Co-crystal structure of *S*. Typhi PltB homopentamer bound to 4-*O*-acetylated α2–3 sialosides. **A,** Crystal structure of PltB homopentamer in complex with Neu4,5Ac_2_α2-3Galβ1-4GlcNAc is shown as a ribbon cartoon with each protomer depicted in a different color. Cyan sticks, sugar carbon atoms. Blue sticks, nitrogen atoms. Red sticks, oxygen atoms. BS1-3, binding sites 1–3. **B,** Surface charge distribution of the PltB pentamer structure and the indicated glycan. **C-D,** Close-up view of the interface between the BS1 (C)/ BS2 (D) and the indicated glycans with their electron density maps. Green sticks, sugar carbon atoms of the 4-*O*-acetyl group. **E-F,** Close-up views of the BS1 (E)/ BS2 (F) complexed with Neu4,5Ac_2_α2-3Galβ1-4GlcNAc. Dotted lines, H-bonds. Gray balls, water.

Next, to investigate whether the O-acetylation at C-9 is important for the increased interactions between PltB and 9-*O*-acetylated α2–3 sialosides, we carried out SPR analysis (Fig 3D and 3G) and solved co-crystal structure of PltB subunits bound to 4-*O*-acetylated counterpart, Neu4,5Ac_2_α2-3Galβ1-4GlcNAc (Fig 6A and 6B and Table 1). *O*-acetylation of Neu5Ac at C-4 position naturally occurs, although it does not appear to occur in humans. Similar to the results with 9-*O*-acetylated α2–3 sialosides, we found that PltB binds to 4-*O*-acetylated α2–3 glycans (Neu4,5Ac_2_α2-3Galβ1-4GlcNAc) with a ~11-fold increased affinity compared to its unmodified α2–3 counterpart (Neu5Acα2-3Galβ1-4GlcNAc) (Fig 3B, 3D and 3G). We also found that 4-*O*-acetylated α2–3 sialosides bind to PltB homopentamer via the BS1 and 2 (Fig 6A–6D). The 4-*O*-acetyl group forms H-bonds in both BS1 and 2 with Tyr33 (via the main chain) and Thr109, respectively (Fig 6E and 6F). These results indicate that the acetylation position at C-9 itself is not important for the increased binding affinities (Fig 3B, 3C, 3D and 3G). However, it indicates that whether the acetyl group of modified glycan receptor moieties contributes to additional interactions in the binding pockets is important for the binding affinity changes (Figs 3, 4 and 6).

We also solve co-crystal structures of PltB homopentamer bound to Neu5,9Ac_2_α2-6Galβ1-4GlcNAc and compare the glycan-binding interface with that for unmodified glycan counterparts. Both unmodified and 9-*O*-acetylated α2–6 glycans bind only to the BS1 (Fig 5A–5E). Notable differences between the two are H-bonds between the 9-*O*-acetyl group and Thr65 (via the main chain), as well as between GlcNAc and Lys59 in the interface for 9-*O*-acetylated α2–6 glycans (Fig 5D and 5E). These results are in support of the increased binding affinity of PltB to 9-*O*-acetylated α2–6 sialosides, compared to unmodified α2–6 counterpart (Fig 3E, 3F and 3G).

### The role of 9-*O*-acetyl modification in typhoid toxin-mediated intoxication

**Fig 7:**
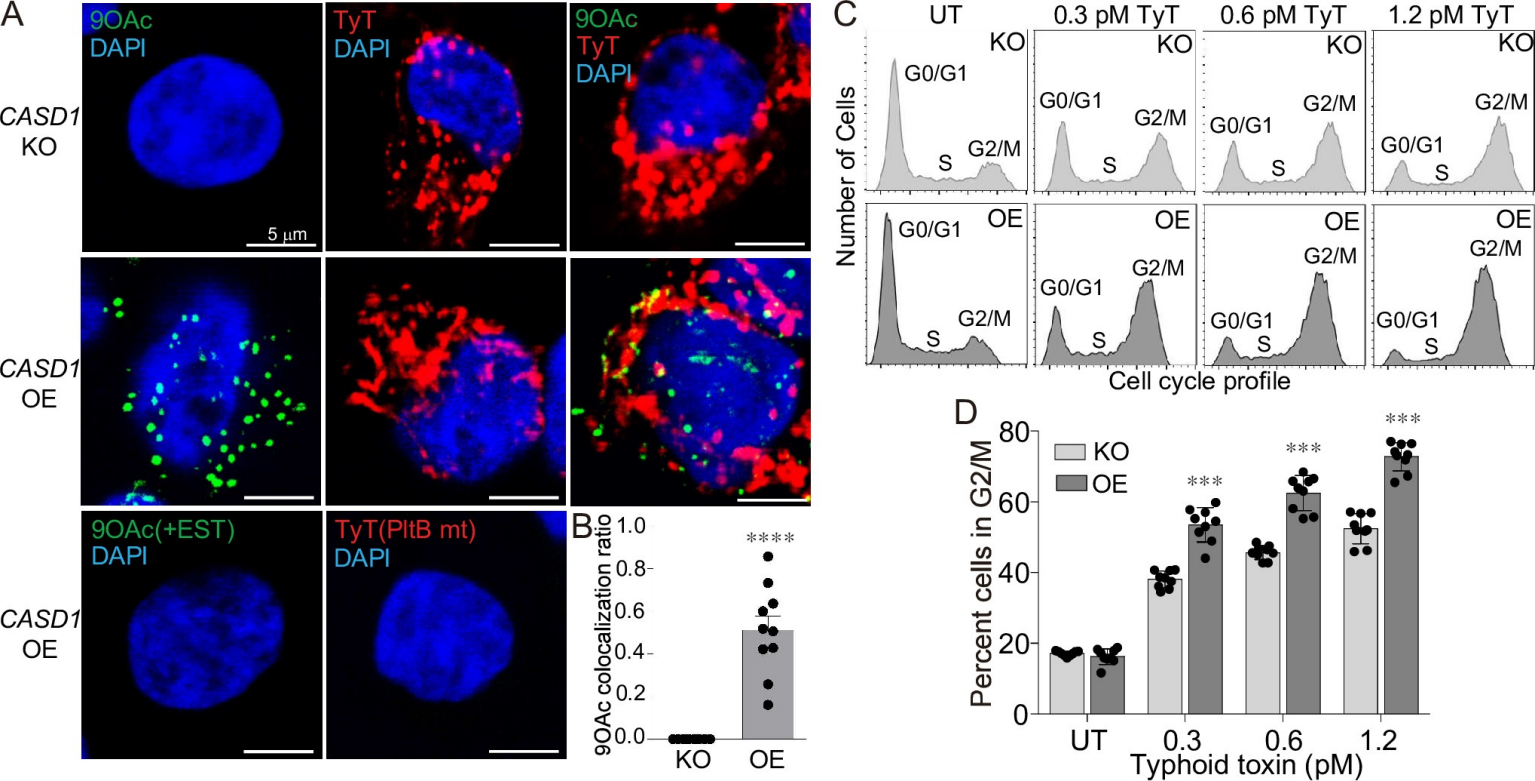
The role of 9-*O*-acetyl modification in typhoid toxin intoxication. **A,** Representative confocal fluorescence microscopy images of the HEK293 *CASD1* KO (top panels) and OE (middle panels) cells stained with PToV-P4 HE-Fc (left; to detect 9-*O*-acetylated Neu5Ac), typhoid toxin (middle; to detect all Neu5Ac), or both PToV-P4 HE-Fc and typhoid toxin (right). DAPI was used to stain DNA (blue). PToV-P4 HE-Fc + esterase activity (EST) and typhoid toxin PltB^S35A^ mutant (a glycan binding-defective mutant) were used to demonstrate the specificity of fluorescent signals (bottom panels). Scale bar, 5 μm. **B,** Co-localization frequency of cells that are positive for both PToV-P4 HE (9-*O*-Ac Neu5Ac) and TyT (all Neu5Ac) are shown. Total cells that are positive for PToV-P4 HE are expressed as 1. Two independent experiments were performed. Bars represent average ± standard error of the mean. **** p<0.0001. n = 10 per group. Two-tailed unpaired t-tests. **C,** Representative cell cycle histograms used for typhoid toxin-mediated intoxication quantification are shown. The gating strategy used is shown in S1 Fig. **D,** Percent cells in G2/M cell cycle reflecting typhoid toxin-mediated intoxication. Indicated cells were treated with various concentrations of typhoid toxin for 24 hrs, whose cell cycle profiles were analyzed via flow cytometry. Three independent experiments were performed. Bars represent average ± standard error of the mean. *** p<0.001. n = 9 per group. Two-tailed unpaired t-tests.

We next investigated whether the increased binding affinities of typhoid toxin to 9-*O*-acetylated glycan receptor moieties alter intoxication outcomes in host cells. We generated HEK293 cells that do not express 9-*O*-acetylated sialosides and counterpart cells that express 9-*O*-acetylated sialosides, via knocking-out (KO) and overexpressing (OE) the Cas1 domain containing 1 (*CASD1*) gene, respectively. CASD1 is responsible for the addition of the *O*-acetyl group to Neu5Ac [30]. 9-*O*-acetylated sialosides are indeed absent in the *CASD1* KO cells, while present in the cell surface of the *CASD1* OE cells (Fig 7A). The signal for 9-*O*-acetylated Neu5Ac is specific since the signal was removed when the esterase of PToV-P4 HE was functional (+EST) (Fig 7A bottom left panel). PToV-P4 HE possessing the functional esterase activity is capable of cleaving off the complex of PToV-P4 HE and 9-*O*-acetylated glycans from host cells. Both *CASD1* KO and *CASD1* OE cells expressed unmodified Neu5Ac on their cell surface, as detected by typhoid toxin on the non-permeabilized cells (Fig 7A top and middle panels). The specificity of the red signal detected by typhoid toxin was validated by employing a glycan-binding defective mutant of typhoid toxin that contains a S35A point mutation in the PltB subunit [10] (Fig 7A bottom right panel). Up to approximately 80% of 9-*O*-acetylated Neu5Ac on the *CASD1* OE plasma membrane was co-stained with typhoid toxin, which is consistent with the capability of typhoid toxin binding to both unmodified and 9-*O*-acetylated Neu5Ac (Fig 7B). Using cell cycle profile analysis, allowing for objectively comparing the susceptibility of cells to typhoid toxin, we found that both *CASD1* KO and *CASD1* OE cells are susceptible to typhoid toxin, but *CASD1* KO cells were less susceptible to typhoid toxin than *CASD1* OE cells by ~4-fold, as 1.2 pM typhoid toxin-treated *CASD1* KO cells in G2/M was comparable to 0.3 pM toxin-treated *CASD1* OE cells (S1 Fig and Fig 7C and 7D). These results indicate that typhoid toxin binding to 9-*O*-acetylated glycan receptor moieties with increased affinities leads to increased intoxication of cells.

## Discussion

Typhoid toxin binding to specific trisaccharide glycan receptor moieties displayed on cell plasma membranes is the initial step for the typhoid intoxication process. Although the glycan sequence that typhoid toxin recognizes is Neu5Ac-Gal-GlcNAc, typhoid toxin has the highest tropism to cells expressing this glycan motif in the context of multiantennary N-linked glycans, as this interaction results in high-affinity, multivalent bindings. The current study provides important information regarding the capability of typhoid toxin PltB in exploiting the glycan motif with structural differences for virulence. Here we show that typhoid toxin PltB binds to the glycan motif terminated in both unmodified and 9-*O*-acetylated Neu5Ac, both of which result in cell intoxication. However, the binding affinity of PltB homopentamer to 9-*O*-acetylated sialosides is higher than that to their unmodified counterparts, which explains the increased susceptibility of cells expressing 9-*O*-acetylated sialosides.

Co-crystal structure analysis suggests that the shallow nature of the binding site BS1 in PltB homopentamer and that the amino acid residues shaping this binding site are important for this aspect. In fact, typhoid toxin is capable of using not only both unmodified and 9-*O*-acetylated glycan receptor moieties but also both α2–3 or α2–6 linked glycan receptor moieties. This aspect of typhoid toxin cell recognition appears to be part of evolutionary processes for promoting virulence and its role in assisting *S*. Typhi persistent infection, which is supported by observations that its orthologue Javiana toxin PltB, with only three amino acid variations, preferentially binds to α2–3 sialosides but not well to α2–6 sialosides [20].

One reason why typhoid toxin has evolved to possess flexible glycan receptor usage, as long as the trisaccharide motif is present, may be associated with the dynamic life cycle of *S*. Typhi. *S*. Typhi infects both locally in the gastrointestinal tract and systemically in the reticuloendothelial cells, as well as gallbladder epithelial cells over the course of acute and persistent infection. Typhoid toxin is produced exclusively by *S*. Typhi located within host cells during infection. Multiantennary N-linked glycans terminated in the trisaccharide motif are highly expressed in these cells, as well as in cells associated with typhoid toxin-mediated clinical signs. However, their structural makeup is different depending on cell types which can be further altered by infection as well as part of normal cell developmental/activation processes. Typhoid toxin is suggested to contribute to both typhoid disease progress and the establishment of persistent infection by entering into both uninfected and *S*. Typhi-infected cells. One fine-tuning mechanism of typhoid toxin in resulting in different virulence between uninfected cells that are associated with clinical signs such as immune cells and brain endothelial cells of arterioles and *S*. Typhi-infected cells may be associated with the binding affinity difference to structurally different glycan receptor moieties. In support of this postulate, the binding affinity of PltB to α2–6 sialosides is higher than that to α2–3 sialosides; intriguingly, cells associated with typhoid toxin-mediated clinical signs predominantly express α2–6 sialosides and cells associated with infection predominantly express α2–3 sialosides.

Typhoid toxin binds to 9-*O*-acetylated glycans with increased affinities, as compared to the affinities to unmodified counterparts. This effect is greater between unmodified and 9-*O*-acetylated α2–3 sialosides. Typhoid toxin binding affinity to 9-*O*-acetylated α2–3 sialosides is comparable to its binding affinity to α2–6 sialosides. Interestingly, this correlates to the absence or the low expression of 9-*O*-acetylated α2–3 glycan receptors in epithelial cells and macrophages, cells associated with *S*. Typhi infection. It is intriguing to speculate that the expression of glycan receptor moieties with relatively lower affinities, in conjunction with the terminally differentiated cell stage in the case of macrophages render cells harboring *S*. Typhi less susceptible to typhoid toxin intoxication process, as compared to other immune cells and brain endothelial cells of arterioles. Humans have evolved to display a different level of 9-*O*-acetylated sialic acids on the surface of various cells and tissues, presumably for the benefits of host cells, such as fine-tuning of immune responses and cell longevity, among others [23, 31, 32]. In support of this notion, host cells displaying *O-*acetylated sialic acids are shown to be more resistant against the sialidase activity, indicating the protective role of *O-*acetyl modification against various pathogens and other cell death stimuli. In line with the observations, longer-lived cells display higher 9-*O-*acetylation levels than shorter-lived cells (e.g., T cells vs. neutrophils or brain vs. other tissues). In this perspective, it is quite remarkable that bacterial exotoxin secreted by the human-adapted pathogen *S*. Typhi has evolved to take advantage of the evolution that occurred to humans, which appears to be another example of arms-race between the host and pathogens.

We demonstrated that the *O*-acetylation position in C-9 itself is not essential but whether the *O*-acetyl group results in increased interactions with residues in the PltB binding sites is important for the increased affinities between PltB and *O*-acetylated glycan receptors. Overall, this finding, along with other information that we uncover in this study, offers useful insight into the development of competitive inhibitors interfering with the interaction between typhoid toxin and endogenous glycan receptors of cells. Also importantly, this study serves as the first example demonstrating that bacterial pathogens or toxins exploit glycan receptors that are modified by adding chemical groups to fine-tune their intoxication outcomes.

## Materials and methods

### Ethics statement

The inclusion of six human volunteers was conducted according to protocols approved by Cornell University’s Institutional Review Board for Human Participants. Peripheral blood draw was performed (to obtain primary peripheral blood leukocytes) by a nurse practitioner at the Cornell Human Metabolic Research Unit. The data were analyzed anonymously. All adult subjects provided informed consent. The written consent form was given to all participants. Mouse tissues were obtained after sacrificing animals, in accordance with protocols approved by Cornell University’s institutional Animal Care and Use Committee. Protocol #2014–0084 assigned by the IACUC/ethics committee that approved my animal experiments. The experiments followed IACUC and AAALAC guidelines.

### Glycan expression profiling of human peripheral blood leukocytes (PBLs)

Blood samples were collected in tubes coated with an anticoagulant via venipuncture. PBLs were prepared by lysing erythrocytes through incubation of blood in ammonium-chloride-potassium (ACK) lysis buffer (Lonza) and stained using a premix antibody cocktail containing various human antibodies as indicated in the gating procedure, along with either Fluorescein-MAL-1 (Vector Labs) or Fluorescein-SNA (Vector Labs). The stained cells were read using a BD FACS Canto II flow cytometry system (BD Biosciences) and analyzed using FlowJo software. PBLs were gated based on their cell surface staining profiles, CD3^+^ (anti-human CD3 conjugated to PE-Cy7, clone SK7, BD Biosciences), CD19^+^ (anti-human CD19 conjugated to PE, clone HIB19, BD Biosciences), CD15^+^CD16^+^ (anti-human CD15 conjugated to APC, clone HI98, BD Biosciences; anti-human CD16 conjugated to APC-Cy7, clone 3G8, BD Biosciences), and CD14^+^ (anti-human CD14 conjugated to PerCP-Cy5.5, clone M5E2, BD Biosciences) for T cells, B cells, neutrophils, and monocytes/macrophages, respectively.

### Glycan expression profiling of CMAH null mouse tissues and cells

The mice were anesthetized with isoflurane, followed by sacrifice-perfusion, which was conducted by sequentially administering 10% sucrose and 4% paraformaldehyde using a pressure-controlled Perfusion One system (Leica Biosystems). After perfusion, the indicated mouse tissues were extracted and fixed in 4% paraformaldehyde for 24 h at 4 °C. Tissues were washed with PBS, immersed in a solution containing 30% sucrose, and incubated overnight for cryoprotection. The tissues were trimmed into coronal sections and placed in cassettes for Tissue-Tek OCT embedding. The embedded tissues were flash-frozen in isopentane cooled to –80 °C. Cryosections of frozen tissue samples were cut to be 8 μm thick and stored in −80 °C until staining.

The frozen tissue sections were incubated with 0.1% Tween 20/3% BSA/PBS for 1 hr at 4°C, followed by incubation with typhoid toxin conjugated to Alexa Fluor 555 and PToV-P4 HE-Fc pre-complexed with Alexa Fluor 488 labeled anti-human IgG antibody to detect Neu5Ac and 9-*O*-acetylated Neu5Ac respectively for 2 h at room temperature or overnight at 4 °C. The sections were washed with PBS and counterstained with 4′,6-diamidino-2-phenylindole (DAPI) for DNA. The slides were mounted in ProLong antifade mounting solution (Molecular Probes, Thermo Fisher Scientific). Digital photomicrographs were taken using a Leica DMI6000B/DFC340 FX fluorescence microscope system. The fluorescent signal intensity of images was quantified using the measure function of ImageJ (National Institutes of Health, USA) after subtracting the background. The colocalization frequency of typhoid toxin (Neu5Ac) and PToV-P4 HE (9-*O*-Ac Neu5Ac) was objectively determined using ImageJ with JACoP plug-in from Manders's overlapping coefficients (fraction of red signal overlapping green signal). Recorded images were merged using the ImageJ merge channels function and processed further with Adobe Photoshop to adjust the brightness and contrast equally for all recordings.

### Surface plasmon resonance (SPR)

***Protein and glycan preparations*:** C-terminally hexahistidine tagged PltB was purified as described previously [19]. Neu5Acα2-3Galβ1-4GlcNAc and Neu5Acα2-6Galβ1-4GlcNAc were purchased from Carbosynth. 9-*O*-Acetylated derivatives of those glycans were synthesized using one-pot three-enzyme sialylation systems in the Xi Chen laboratory [22]. In brief, sialosides containing 9-*O*-acetylated Neu5Ac (Neu5,9Ac_2_α2-3Galβ1-4GlcNAcβProN_3_ and Neu5,9Ac_2_α2-6Galβ1-4GlcNAcβProN_3_), and 4-*O*-acetylated Neu5Ac (Neu4,5Ac_2_α2-3Galβ1-4GlcNAcβProN_3_) were synthesized from Galβ1-4GlcNAcβProN_3_ (LacNAcβProN_3_) [33] as described previously using Neu5,9Ac_2_ [34] and Neu4,5Ac_2_ [35], respectively, as the donor precursors in an one-pot two-enzyme sialylation system [36] containing *Neisseria meningitidis* CMP-sialic acid synthetase (NmCSS) [37] and a sialyltransferase. *Pasteurella multocida* α2–3 sialyltransferase 1 M144D (PmST1 M144D) mutant [38] was used for the synthesis of Neu5,9Ac_2_α2-3Galβ1-4GlcNAcβProN_3_ and *Photobacterium sp*. α2–6 sialyltransferase A366G (Psp26ST A366G) [39] was used for synthesizing Neu5,9Ac_2_α2-6Galβ1-4GlcNAcβProN_3_ while *P*. *multocida* α2–3 sialyltransferase 3 (PmST3) [40] was used for synthesizing Neu4,5Ac_2_α2-3Galβ1-4GlcNAcβProN_3_) [35].

***SPR*:** Stock solutions of 100 mM of glycans in 10 mM HEPES, pH 7.4 and 150 mM NaCl were prepared for each glycan. SPR experiments were conducted using OpenSPR (Nicoya) with all system buffers filtered through 0.2-micron filters. To immobilize PltB pentamer on sensor chips, we carried out sequentially the following steps. We first loaded NTA conjugated colloidal gold sensor chip on OpenSPR machine, equilibrated with a buffer containing 10 mM HEPES, pH 7.4 and 150 mM NaCl (running buffer) and maintained the system under a continuous flow of 100 μL/min. The chip was then activated with 150 μL of 200 mM of Imidazole followed by 150 μL of 15 mM Tris-HCl pH 8.0, 150 mM NaCl and 50 mM EDTA. Cobalt ion was introduced onto the chip, after an initial injection of 150 μL of Milli-Q water, through an injection of 150 μL of 20 mM cobalt chloride, followed by a wash with 150 μL of Milli-Q water and 150 μL of running buffer. PltB pentamer was loaded onto the sensor chip through an injection of 150 μL of 100 μg/mL of purified protein and washed twice with 150 μL of running buffer. Subsequently, 150 μL of serially diluted glycans was then introduced with an injection of 150 μL of running buffer in-between glycan injections. For α-2,3 unmodified glycan, the concentrations tested are: 10 mM, 5 mM, 2.5 mM, 1.25 mM and 0.625 mM. For all other glycans, the concentrations tested are: 2.5 mM, 1 mM, 0.5 mM, 0.25 mM, 0.125 mM. Sensograms were collected for at least two minutes per each injection. Collected sensograms were analyzed by Tracedrawer software (Ridgeview Instruments AB) and fitted with kinetic parameter curves using the provided one-to-one formulation.

### Crystallization

Purified C-terminal hexahistidine-tagged PltB were prepared as described previously and used for crystallization [19]. Optimized hanging drop crystallization, glycan soak, and cryo-protection conditions were reproduced as previously described [20] (Table 2). Matured crystal appeared after one day and was soaked with Neu5,9Ac_2_α2-3Galβ1-4GlcNAc, Neu4,5Ac_2_α2-3Galβ1-4GlcNAc, and Neu5,9Ac_2_α2-6Galβ1-4GlcNAc separately. The crystals were cryoprotected with 20% ethylene glycol and flash-frozen in liquid nitrogen upon harvest.

**Table 2:** Related to Figs 4–6. Crystallization and glycan soaking conditions for *S*. Typhi PltB homopentamer.

PDB ID	Protein	Conc. (mg/ml)	Crystallization condition	Glycan	Soaking condition	Cryo-protection
6TYN	PltB	5.1	26% PEG1500, Sodium acetate pH 4.4	Neu5,9Acs α2–3 Gal β1–4 GlcNAc	5 mM, 30 min, RT	20% Ethylene glycol
6TYO	PltB	5.1		Neu4,5Acs α2–3 Gal β1–4 GlcNAc	5 mM, 30 min, RT	20% Ethylene glycol
6TYQ	PltB	5.1		Neu5,9Acs α2–6 Gal β1–4 GlcNAc	3 mM, 2hr, RT	20% Ethylene glycol

### X-ray data collection and structure determination

X-ray diffraction for PltB crystals soaked with Neu5,9Ac_2_α2-3Galβ1-4GlcNAc and Neu4,5Ac_2_α2-3Galβ1-4GlcNAc were obtained through the GMCA APS (www.gmca.aps.anl.gov) 23-ID-B beamline at an incident beam of 1.87 Å in wavelength and 100K temperature. The resulting PltB diffraction data from single crystals were processed using HKL-2000 [41]. X-ray diffraction for PltB crystals soaked with Neu5,9Ac_2_α2-6Galβ1-4GlcNAc was obtained through the NE-CAT (lilith.nec.aps.anl.gov) APS 24-ID-C synchrotron beamline at an aperture of 30 **μ**m at 5% beam strength and cryo-cooled to 100K. The resulting diffraction data from a single crystal were processed using RAPD [42, 43]. All structures were phased using the molecular replacement method in PHENIX [44] using previously published PltB apo structure (PDB: 4RHR) as a search model [19]. The bound glycans were fitted into density within the soaked crystal structures. Rebuilding and real-space refinement were performed with Coot [45] together with reciprocal space refinements in PHENIX and validation in MolProbity [46]. Figures were prepared using PyMol (Schrodinger Inc). The data collection and refinement statistics are summarized in Table 1. Coordinates for the atomic structures have been deposited in the RCSB Protein Data Bank under PDB numbers 6TYN, 6TYO, and 6TYQ.

### Knockout and overexpression of *CASD1* in HEK293 cells

*CASD1* knockout (KO) and overexpression HEK293 cells were generated in the Colin Parrish laboratory. Nickase Cas9 plasmids (PX462, Addgene plasmid #62897) were used to target an adjacent site in the early exons of *CASD1*. The target site used was 5’-TCAACCACTACTTCAGCGTGAGG. Transfected cells were selected with puromycin and single-cell clones screened with PToV-P4 HE-Fc to identify non-staining variants. Edited sequences were confirmed by PCR amplification of the targeted regions, and sequencing the PCR product for each allele. KO cell lines were used to prepare overexpression cell lines by transfection of a pcDNA3.1(-) plasmid expressing the complete human *CASD1* cDNA open reading frame synthesized by Bio Basic (Markham, Ontario, Canada). Transfected cells were selected with G418 and single-cell clones screened by staining with PToV-P4 HE-Fc to identify 9-*O*-Ac positive cell lines. Full sequencing of each allele and qPCR were performed to confirm the deletion of the gene.

### Cell intoxication assay

Host cell cycle profile quantification via flow cytometry as previously described [10, 11, 19]. Briefly, after the treatment of cells with typhoid toxin for 24 hrs with indicated concentrations, cells were trypsinized, harvested, washed, and fixed for 2 hours at -20°C in a buffer containing 70% ethanol in PBS. Fixed cells were washed with PBS for 2 times and resuspended in 500 **μ**L of PBS containing 50 **μ**g/ml propidium iodide, 100 **μ**g/ml RNase A, and 0.05% Triton X-100. After incubation for 40 min at 37°C, cells were washed with PBS, resuspended in 200 **μ**L PBS, filtered, and analyzed via flow cytometry. DNA contents of cells were determined using FlowJo software (Treestar).

### Statistical analysis

The p values were calculated using a two-tailed, unpaired Student’s t-test for two-group comparisons in GraphPad Prism (GraphPad Software) unless otherwise specified. P values <0.05 were considered significant.

## Supporting information

S1 Movie This movie shows a series of structure alignment data in a following order, which is also indicated in the movie: (1) typhoid toxin (PDB number 4K6L), (2) typhoid toxin (grey; PDB number 4K6L) with PltB pentamer bound to Neu5Acα2-3Galβ1-4GlcNAc (blue; PDB number 6P4M), (3) typhoid toxin with PltB bound to Neu5Acα2-6Galβ1-4GlcNAc (red; PDB number 6P4N), (4) typhoid toxin with PltB bound to Neu5,9Ac_2_α2-3Galβ1-4GlcNAc (magenta; PDB number 6TYN), (5) typhoid toxin with PltB bound to Neu5,9Ac_2_α2-6Galβ1-4GlcNAc (cyan; PDB number 6TYQ), and (6) typhoid toxin with PltB bound to Neu4,5Ac_2_α2-3Galβ1-4GlcNAc (orange; PDB number 6TYO).
(MP4)

S1 Fig Representative flow cytometric analysis of cell cycle profiles. Doublets and multiplets, as well as cell debris, were gated out from the total population (left panel) and cell cycle profiles of singlets were analyzed (right panel).
(TIF)
