# Supplementary figures and images for: The role of 9-O-acetylated glycan receptor moieties in the typhoid toxin binding and intoxication
Source: PLoS Pathog. 2020 Feb 21;16(2):e1008336. doi: 10.1371/journal.ppat.1008336 (PMC7055914; doi:10.1371/journal.ppat.1008336)

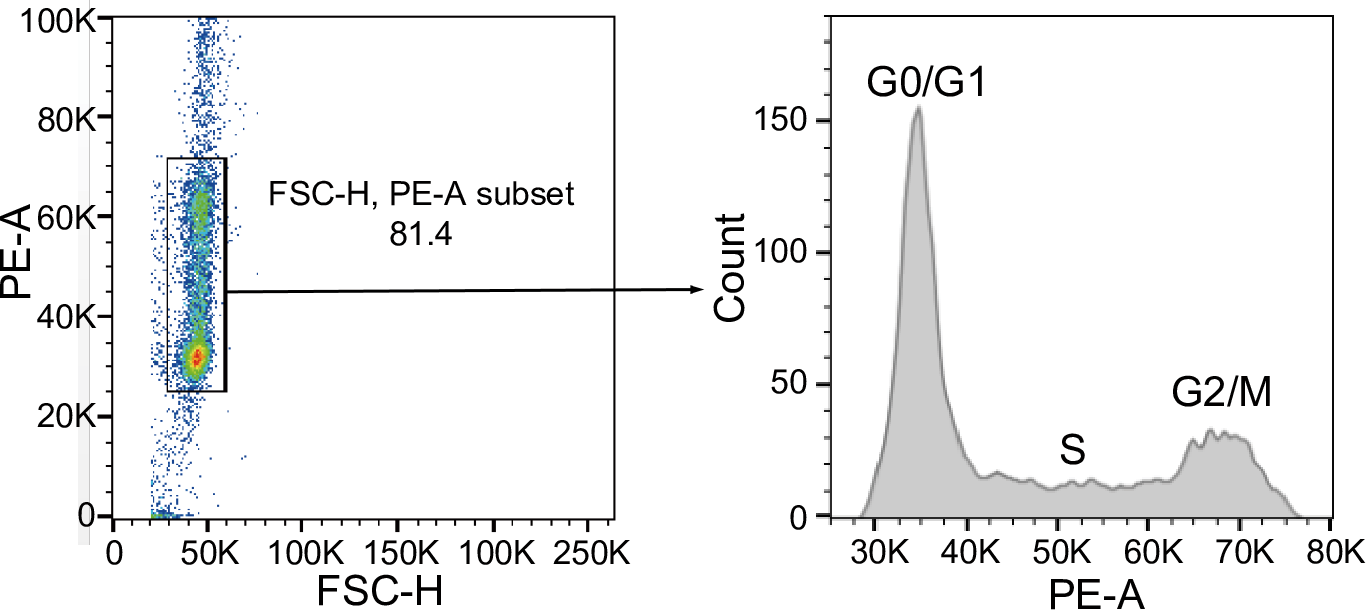

Supplement: S1 Fig — Doublets and multiplets, as well as cell debris, were gated out from the total population (left panel) and cell cycle profiles of singlets were analyzed (right panel). (TIF) [file ppat.1008336.s002.tif]
